# Machine Learning for the Prediction of Synchronous Organ-Specific Metastasis in Patients With Lung Cancer

**DOI:** 10.3389/fonc.2022.817372

**Published:** 2022-05-13

**Authors:** Huan Gao, Zhi-yi He, Xing-li Du, Zheng-gang Wang, Li Xiang

**Affiliations:** ^1^ School of Medicine and Health Management, Huazhong University of Science and Technology, Wuhan, China; ^2^ Tongji Hospital, Tongji Medical College, Huazhong University of Science and Technology, Wuhan, China

**Keywords:** machine learning, artificial neural network, SEER, metastasis, lung cancer

## Abstract

**Background:**

This study aimed to develop an artificial neural network (ANN) model for predicting synchronous organ-specific metastasis in lung cancer (LC) patients.

**Methods:**

A total of 62,151 patients who diagnosed as LC without data missing between 2010 and 2015 were identified from Surveillance, Epidemiology, and End Results (SEER) program. The ANN model was trained and tested on an 75/25 split of the dataset. The receiver operating characteristic (ROC) curves, area under the curve (AUC) and sensitivity were used to evaluate and compare the ANN model with the random forest model.

**Results:**

For distant metastasis in the whole cohort, the ANN model had metrics AUC = 0.759, accuracy = 0.669, sensitivity = 0.906, and specificity = 0.613, which was better than the random forest model. For organ-specific metastasis in the cohort with distant metastasis, the sensitivity in bone metastasis, brain metastasis and liver metastasis were 0.913, 0.906 and 0.925, respectively. The most important variable was separate tumor nodules with 100% importance. The second important variable was visceral pleural invasion for distant metastasis, while histology for organ-specific metastasis.

**Conclusions:**

Our study developed a “two-step” ANN model for predicting synchronous organ-specific metastasis in LC patients. This ANN model may provide clinicians with more personalized clinical decisions, contribute to rationalize metastasis screening, and reduce the burden on patients and the health care system.

## Introduction

Lung cancer (LC) is one of the most commonly diagnosed malignancy as well as the leading cause of cancer-related death both in males and females worldwide (1, 2). Approximately 30-40% of LC patients present with distant metastasis (DM) at the time of diagnosis (3–5). And distant metastasis is responsible for a large morbidity and mortality burden among LC patients (6, 7). The most common metastatic site is bone, followed by liver, brain and adrenal gland (8, 9). Distant metastasis is closely related to treatment decisions and clinical outcomes. Therefore, it is important to identify and diagnose distant metastasis in the early period.

Computed tomography (CT), magnetic resonance imaging (MRI), single-photon emission computed tomography (SPECT) and positron emission tomography/computed tomography (PET/CT) are the common techniques to screen the distant metastasis in LC patients. However, routine DM screening to all LC patients is controversial because of low detection rate of asymptomatic patients, invasive operation, potential risk of adverse reactions, complex process and high cost (10–14). Therefore, there are strong requirements for the identification of a high-risk group with distant metastasis and the rationalization of DM screening in LC patients.

The occurrence and development of lung cancer is very complicated, and most of the clinical characteristics exhibit a multidimensional and non-linear relationship. The artificial neural network (ANN) is a complex non-linear model inspired by the working of biological neural networks (15–17). In the face of huge and complex medical data, it has the ability to discover underlying patterns and constantly adjust the algorithm to adapt to new patient information (18–20). In recent years, the ANN has been applied successfully in clinical medicine, including diagnosis, image identification and outcome prediction (16, 21–24).

In this study, we aim to develop an ANN model to predict synchronous organ-specific metastasis in LC patients. This study may provide clinicians with more personalized clinical decisions, reduce the unnecessary financial burden of patients, and allocate medical resources more rationally.

## Patients and Methods

### Patient Selection and Data Collection

We obtained the research participants from the Surveillance, Epidemiology, and End Results (SEER) Program. The SEER program is supported by the US National Cancer Institute, covers cases from 18 cancer registries, and represents approximately 28-30% of the population (25). Patient data were screened *via* the SEER*Stat software (version 8.3.6). Since the data was anonymized, no additional institutional review board approval or patient informed consent was required.

We included patients diagnosed with lung cancer between 2010 and 2015. Variables of interest included age, sex, race, marital status, insurance, primary site, histology, grade, tumor size, separate tumor nodules, visceral pleural invasion, T-stage, N-stage, and organ-specific metastases. We excluded the patients whose reporting sources were “Autopsy only” or “Death certification only”, as well as those who did not have complete information on all the above variables.

### Model Development

**Figure 1:**
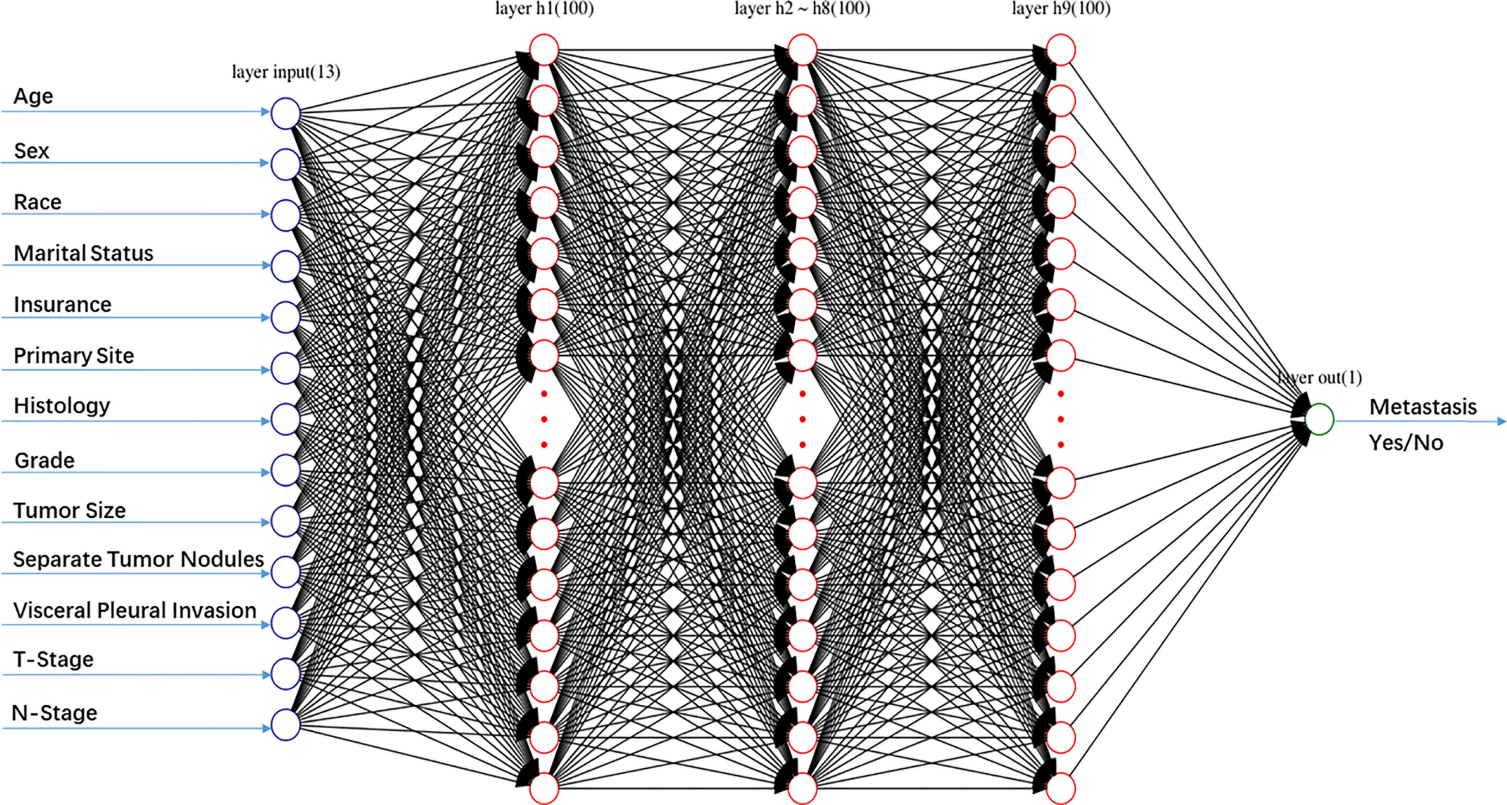
Schematic structure of the artificial neural network (ANN) model including one input layer with 13 nodes, nine hidden layers with 100 nodes, and one output layer with 1 node.

A multilayer perceptron ANN was created consisting of an input, an output, and one or more hidden layers (**Figure 1**). In this research, thirteen selected demographic or clinical variables were served as the input layers neurons, and one variable (metastasis or no metastasis) was served as the output layer neuron. The number of neurons in the hidden layer was set empirically. 75% of patients was used to develop the model (the training group), while the remaining 25% was used to evaluate the developed model (the testing group). A back propagation (BP) method was used to train the multilayer perceptron ANN, which modified the weight of the interneuron connections to reduce the total errors during the repeated development cycles. During the learning progresses, the errors between ANN model outputs and expected outputs were minimized (21). In this study, the number of epochs was selected from the set {10, 20, 30, 50, 100, 500}.

### Statistical Analysis

Kaplan-Meier analysis was used for comparison of survival among the subgroups classified by distant metastasis. Multivariate Cox proportional hazard analyses was conducted to estimate the hazard ratio (HR), and the corresponding 95% confidence interval (CI), for the potential risk factors. The model performance was evaluated with the receiver operating characteristic (ROC) curves and areas under the curve (AUC), which is a score ranging from 0.50 to 1.0. All statistical analyses were conducted using SPSS version 21.0 and RStudio Version 1.0.153. A two-tailed P value <0.05 was considered statistically significant.

## Results

### Patient Demographics and Clinical Characteristics

From 2010 to 2015, 62,151 patients with lung cancer were consecutively included in this study. Patient characteristics were described in **Table 1**. The population with a median age of 68 (IQR, 61-75) years and White people (n=50589, 81.4%) predominated. The distribution of male and female was almost 1:1. The most common primary site was upper lobe (n=37284, 60%) and the most common histological subtype was adenocarcinoma (n=33036, 53.2%). Of these patients, 12,182 (19.6%) developed distant metastases, including 3,982 (6.4%) with bone metastases, 3,674 (5.9%) with brain metastases, and 1,307 (2.1%) with liver metastases.

**Table 1:** Baseline demographic and clinical characteristics of patients with lung cancer.

Characteristics	Total patients	Patients with no metastasis	Patients with metastases	Patients with bone metastasis	Patients with brain metastasis	Patients with liver metastasis
	n=62151	n=49969	n=12182	n=3982	n=3674	n=1307
**Age, year**						
Mean±SD	68±11	68+10	66+11	68±11	64±10	68±10
Median	68	68	66	68	64	68
(IQR 25%-75%)	(61-75)	(61-76)	(59-74)	(60-76)	(57-72)	(61-76)
**Sex**						
Male	31736 (51.1%)	24926 (49.9%)	6810 (55.9%)	2333 (58.6%)	1904 (51.8%)	724 (55.4%)
Female	30415 (48.9%)	25043 (50.1%)	5372 (44.1%)	1649 (41.4%)	1770 (48.2%)	583 (44.6%)
**Race**						
White	50589 (81.4%)	40911 (81.9%)	9678 (79.4%)	3134 (78.7%)	2885 (78.5%)	1076 (82.3%)
Blake	6855 (11%)	5326 (10.7%)	1529 (12.6%)	526 (13.2%)	491 (13.4%)	169 (12.9%)
American Indian/Alaska Native	291 (0.5%)	241 (0.5%)	50 (0.4%)	17 (0.4%)	18 (0.5%)	4 (0.3%)
Asian or Pacific Islander	4416 (7.1%)	3491 (7%)	925 (7.6%)	305 (7.7%)	280 (7.6%)	58 (4.4%)
**Marital Status**						
Single (never married)	8840 (14.2%)	6834 (13.7%)	2006 (16.5%)	605 (15.2%)	700 (19.1%)	199 (15.2%)
Married (including common law)	34269 (55.1%)	27547 (55.1%)	6722 (55.2%)	2235 (56.1%)	1941 (52.8%)	683 (52.3%)
Separated	726 (1.2%)	577 (1.2%)	149 (1.2%)	49 (1.2%)	43 (1.2%)	21 (1.6%)
Divorced	8267 (13.3%)	6637 (13.3%)	1630 (13.4%)	499 (12.5%)	525 (14.3%)	179 (13.7%)
Widowed	10049 (16.2%)	8374 (16.8%)	1675 (13.7%)	594 (14.9%)	465 (12.7%)	225 (17.2%)
**Insurance**						
Uninsured	1602 (2.6%)	1136 (2.3%)	466 (3.8%)	105 (2.6%)	178 (4.8%)	39 (3%)
Insured/Medicaid	60549 (97.4%)	48833 (97.7%)	11716 (96.2%)	3877 (97.4%)	3496 (95.2%)	1268 (97%)
**Primary Site**						
Main bronchus	2036 (3.3%)	1388 (2.8%)	648 (5.3%)	196 (4.9%)	154 (4.2%)	103 (7.9%)
Upper lobe	37284 (60%)	29918 (59.9%)	7366 (60.5%)	2437 (61.2%)	2324 (63.3%)	740 (56.6%)
Middle lobe	3136 (5%)	2600 (5.2%)	536 (4.4%)	170 (4.3%)	158 (4.3%)	58 (4.4%)
Lower lobe	19008 (30.6%)	15486 (31%)	3522 (28.9%)	1146 (28.8%)	1010 (27.5%)	389 (29.8%)
Overlapping lesion of lung	687 (1.1%)	577 (1.2%)	110 (0.9%)	33 (0.8%)	28 (0.8%)	17 (1.3%)
**Histology**						
Squamous cell carcinoma	17973 (28.9%)	15782 (31.6%)	2191 (18%)	874 (21.9%)	515 (14%)	331 (25.3%)
Small cell carcinoma	3236 (5.2%)	1807 (3.6%)	1429 (11.7%)	244 (6.1%)	339 (9.2%)	341 (26.1%)
Adenocarcinoma	33036 (53.2%)	26471 (53%)	6565 (53.9%)	2229 (56%)	2185 (59.5%)	429 (32.8%)
Large cell carcinoma	1117 (1.8%)	830 (1.7%)	287 (2.4%)	78 (2%)	101 (2.7%)	32 (2.4%)
Adenosquamous carcinoma	5244 (8.4%)	3609 (7.2%)	1635 (13.4%)	532 (13.4%)	518 (14.1%)	161 (12.3%)
Sarcomatoid carcinoma	183 (0.3%)	146 (0.3%)	37 (0.3%)	15 (0.4%)	12 (0.3%)	1 (0.1%)
Carcinoid tumor	1362 (2.2%)	1324 (2.6%)	38 (0.3%)	10 (0.3%)	4 (0.1%)	12 (0.9%)
**Grade**						
Well differentiated	7619 (12.3%)	7183 (14.4%)	436 (3.6%)	170 (4.3%)	111 (3%)	37 (2.8%)
Moderately differentiated	21737 (35%)	18991 (38%)	2746 (22.5%)	1072 (26.9%)	816 (22.2%)	199 (15.2%)
Poorly differentiated	29483 (47.4%)	21774 (43.6%)	7709 (63.3%)	2489 (62.5%)	2406 (65.5%)	785 (60.1%)
Undifferentiated	3312 (5.3%)	2021 (4%)	1291 (10.6%)	251 (6.3%)	341 (9.3%)	286 (21.9%)
**Tumor Size, mm**						
Mean±SD	42±25	39±24	52	51±25	52±25	53±26
Median	35	32	48	46	48	50
(IQR 25%-75%)	(22-56)	(20-52)	(32-69)	(32-67)	(32-68)	(33-70)
**Separate Tumor Nodules**						
STN0	55677 (89.6%)	47096 (94.3%)	8581 (70.4%)	2798 (70.3%)	2788 (75.9%)	945 (72.3%)
STN1	2276 (3.7%)	901 (1.8%)	1375 (11.3%)	445 (11.2%)	365 (9.9%)	145 (11.1%)
STN2	2416 (3.9%)	1187 (2.4%)	1229 (10.1%)	421 (10.6%)	312 (8.5%)	117 (9%)
STN3	1782 (2.9%)	785 (1.6%)	997 (8.2%)	318 (8%)	209 (5.7%)	100 (7.7%)
**Visceral Pleural Invasion**						
PL0	21565 (34.7%)	20633 (41.3%)	932 (7.7%)	278 (7%)	338 (9.2%)	101 (7.7%)
PL1	1758 (2.8%)	1715 (3.4%)	43 (0.4%)	5 (0.1%)	26 (0.7%)	4 (0.3%)
PL2	1513 (2.4%)	1455 (2.9%)	58 (0.5%)	15 (0.4%)	30 (0.8%)	6 (0.5%)
PL3	686 (1.1%)	648 (1.3%)	38 (0.3%)	18 (0.5%)	12 (0.3%)	2 (0.2%)
PLX	36629 (58.9%)	25518 (51.1%)	11111 (91.2%)	3666 (92.1%)	3268 (88.9%)	1194 (91.4%)
**T-Stage**						
T1a	11271 (18.1%)	10696 (21.4%)	575 (4.7%)	183 (4.6%)	214 (5.8%)	69 (5.3%)
T1b	8238 (13.3%)	7397 (14.8%)	841 (6.9%)	288 (7.2%)	267 (7.3%)	86 (6.6%)
T2a	17176 (27.6%)	14653 (29.3%)	2523 (20.7%)	832 (20.9%)	840 (22.9%)	264 (20.2%)
T2b	5989 (9.6%)	4615 (9.2%)	1374 (11.3%)	400 (10%)	485 (13.2%)	143 (10.9%)
T3	9616 (15.5%)	6763 (13.5%)	2853 (23.4%)	951 (23.9%)	869 (23.7%)	293 (22.4%)
T4	9861 (15.9%)	5845 (11.7%)	4016 (33%)	1328 (33.4%)	999 (27.2%)	452 (34.6%)
**N-Stage**						
NX	626 (1%)	346 (0.7%)	280 (2.3%)	93 (2.3%)	83 (2.3%)	32 (2.4%)
N0	32972 (53.1%)	30260 (60.6%)	2712 (22.3%)	863 (21.7%)	1066 (29%)	281 (21.5%)
N1	6262 (10.1%)	5116 (10.2%)	1146 (9.4%)	386 (9.7%)	386 (10.5%)	120 (9.2%)
N2	17174 (27.6%)	11319 (22.7%)	5855 (48.1%)	1885 (47.3%)	1641 (44.7%)	642 (49.1%)
N3	5117 (8.2%)	2928 (5.9%)	2189 (18%)	755 (19%)	498 (13.6%)	232 (17.8%)

SD, standard deviation; IQR, interquartile range; STN0, no separate tumor nodules noted; STN1, separate tumor nodules in ipsilateral lung, same lobe; STN2, separate tumor nodules in ipsilateral lung, different lobe; STN3, separate tumor nodules, ipsilateral lung, same and different lobe.

### Survival Analysis

**Figure 2:**
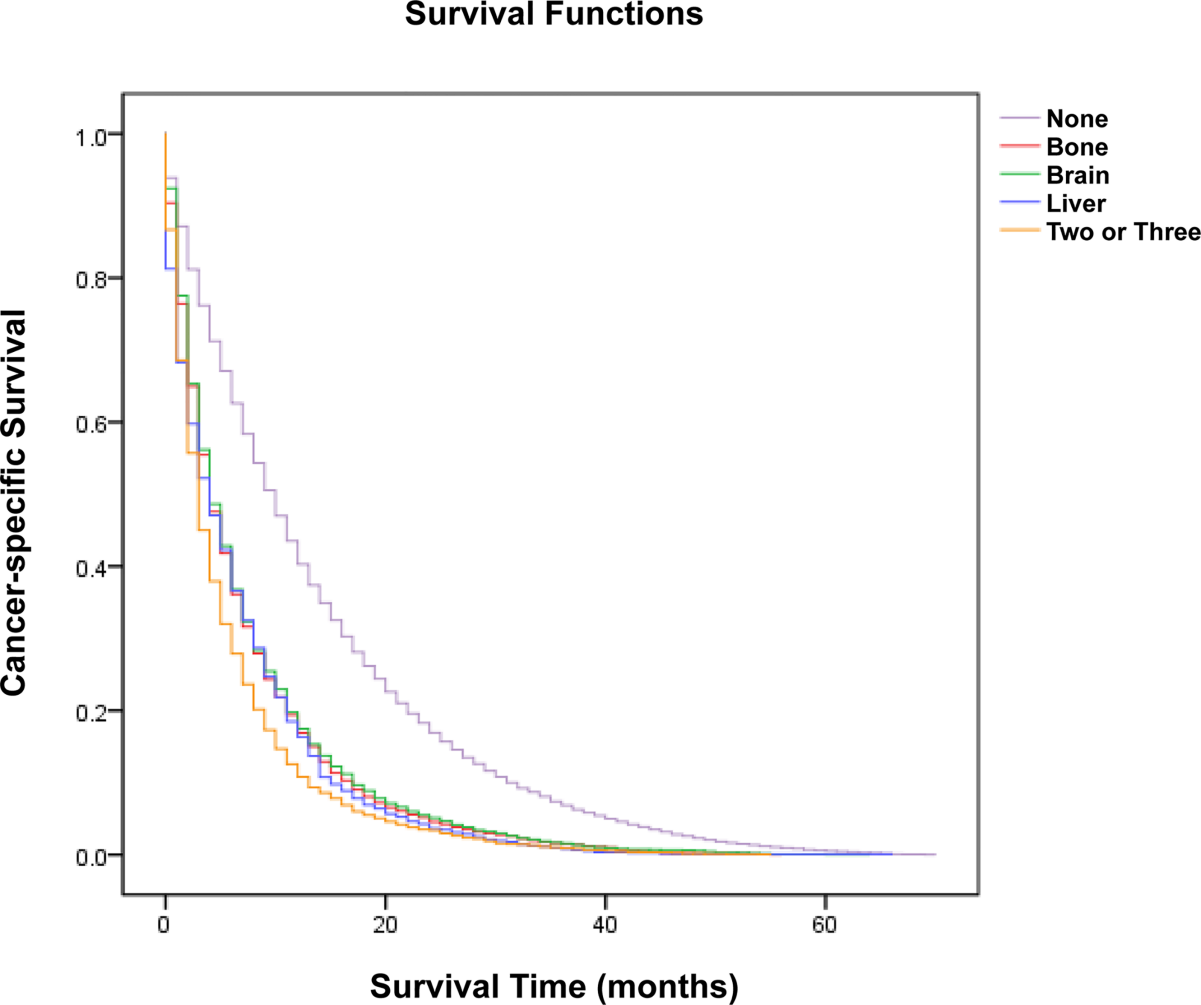
Kaplan-Meier analysis of cancer-specific survival for patients with lung cancer stratified by organ-specific metastasis.

A cohort of 29,296 patients was used to analyze cancer-specific survival (CSS). The median CSS for patients with none metastasis, bone metastasis, brain metastasis, liver metastasis and two or three metastases were 10 months, 4 months, 4 months, 4 months and 3 months, respectively (**Table 2**). Kaplan-Meier analysis showed the similar trend in **Figure 2**. In addition, multivariate Cox proportional hazard analyses revealed that bone metastasis (OR=1.630, p<0.001), brain metastasis (OR=1.698, p<0.001), liver metastasis (OR=1.673, p<0.001) and two or three metastases (OR=2.025, p<0.001) were associated with poor prognosis (**Table 2**).

**Table 2:** Cancer-specific survival and multivariate analysis for patients with lung cancer.

Site	No. (%)	Cancer-specific survival			Multivariate analysis	
		Median	Mean	SD	HR (95% CI)	P-value
None	19139 (65.3)	10	13.4	12.761	1	
Bone	3262 (11.1)	4	6.97	8.061	1.630 (1.568-1.695)	<0.001
Brain	2974 (10.2)	4	7.22	8.4	1.698 (1.631-1.768)	<0.001
Liver	1126 (3.8)	4	6.46	7.63	1.673 (1.573-1.778)	<0.001
Two or Three	2795 (9.5)	3	5.48	7.075	2.025 (1.941-2.112)	<0.001
Total	29296	7	11.03	11.769		

SD, standard deviation; HR, hazard ratio; CI, confidence interval.

### Construction of the ANN Model

**Figure 3:**
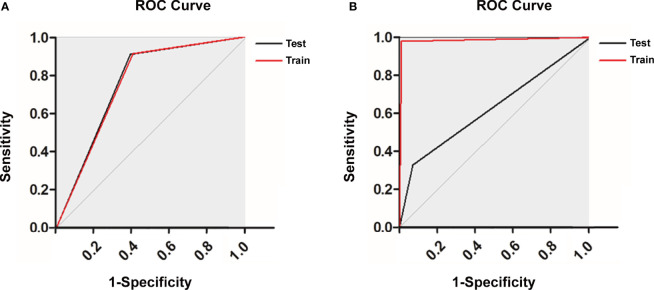
Receiver operating characteristic curve of **(A)** the artificial neural network (ANN) model and **(B)** the random forest (RF) model.

In the training of ANN model, we manually increased the number of hidden layers starting with 5 layers. The predictive sensitivity culminated with 9 layers and adding more layer did not improve the performance but increased time of computation (**Table 3**). In the end, the ANN model was constructed with 13 neurons in the input layer, 100 neurons in each of the 9 hidden layers and 1 neuron in the output layer (**Figure 1**). Meanwhile, we compared the RF model (ntree=500) with the ANN model, and the RF model showed obvious overfitting (**Figure 3**).

**Table 3:** Performance of the artificial neural network (ANN) model with increasing layers for predicting distant metastasis.

Number of the hidden layer	AUC	Sensitivity	Specificity	Accuracy	FPR	FNR	LRP	LRN
5	0.737	0.776	0.697	0.713	0.303	0.224	2.565	0.321
6	0.747	0.815	0.679	0.705	0.321	0.185	2.536	0.273
7	0.748	0.837	0.660	0.691	0.340	0.163	2.460	0.247
8	0.759	0.889	0.629	0.679	0.371	0.111	2.398	0.176
9	0.759	0.906	0.613	0.669	0.387	0.094	2.339	0.154
10	0.761	0.902	0.620	0.674	0.380	0.098	2.371	0.158
11	0.756	0.896	0.609	0.665	0.391	0.104	2.293	0.170

AUC, area under curve; FPR, false positive rate; FNR, false negative rate; LRP, likelihood ratio positive; LRN, likelihood ratio negative.

### Evaluation of the ANN Model

**Figure 4:**
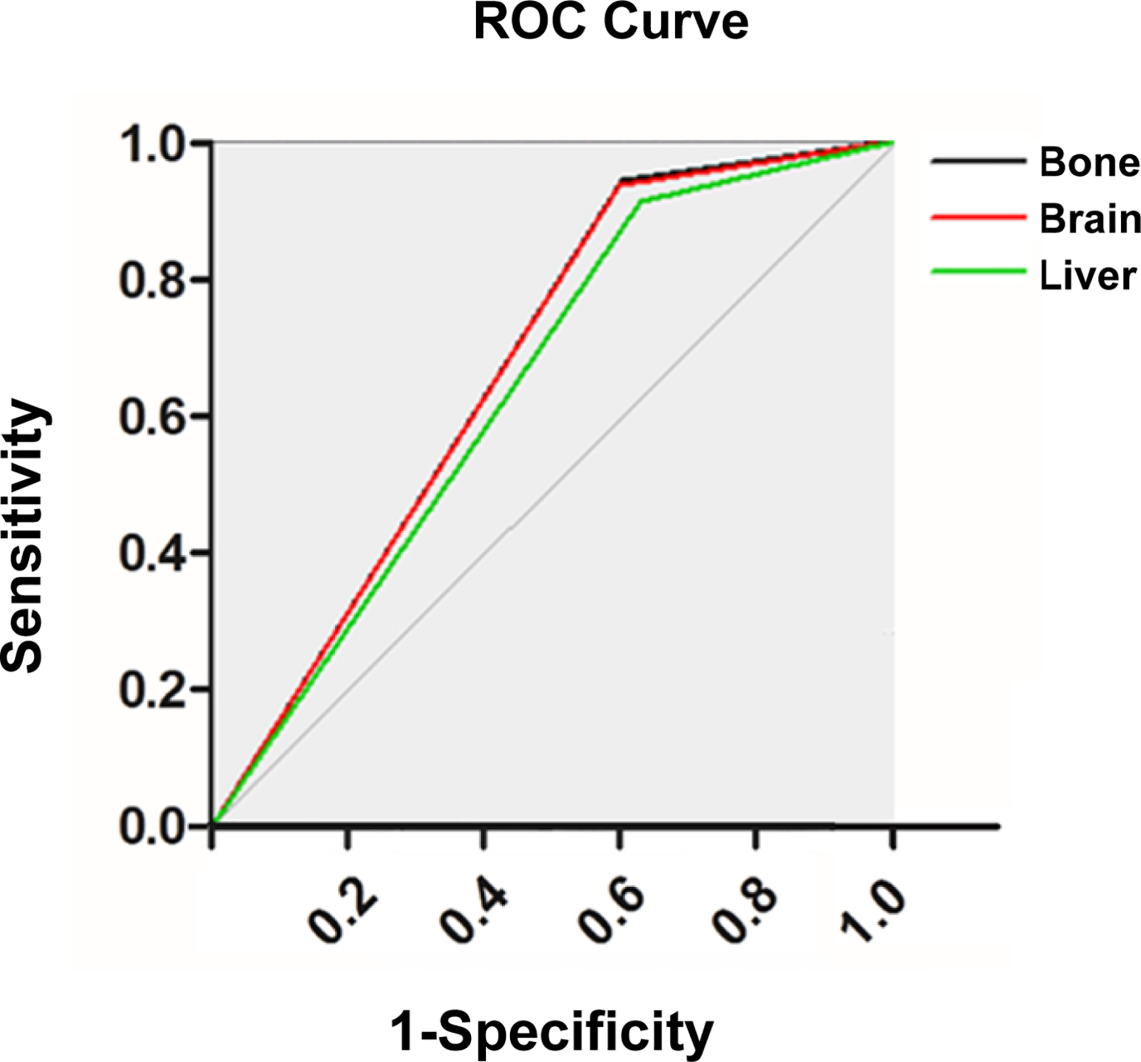
Receiver operating characteristic curve of the artificial neural network (ANN) model for predicting organ-specific metastasis.

In this study, we first evaluated the model performance for predicting distant metastasis in the whole cohort (AUC: 0.759, accuracy: 0.669, sensitivity: 0.906, specificity: 0.613, false positive rate: 0.387, false negative rate: 0.094, likelihood ratio positive: 2.339, likelihood ratio negative: 0.154). Then we evaluated the model performance for predicting organ-specific metastasis in the cohort with distant metastasis (**Figure 4**; **Table 4**). The sensitivity in bone metastasis, brain metastasis and liver metastasis were 0.913, 0.906 and 0.925, respectively.

**Table 4:** Performance of the artificial neural network (ANN) model for predicting organ-specific metastasis.

Site of the organ-specific metastasis	AUC	Sensitivity	Specificity	Accuracy	FPR	FNR	LRP	LRN
Bone	0.688	0.913	0.443	0.539	0.557	0.087	1.638	0.197
Brain	0.686	0.906	0.449	0.525	0.551	0.094	1.646	0.209
Liver	0.664	0.925	0.403	0.453	0.597	0.075	1.548	0.187

AUC, area under curve; FPR, false positive rate; FNR, false negative rate; LRP, likelihood ratio positive; LRN, likelihood ratio negative.

### Variable Importance Measure

**Figure 5:**
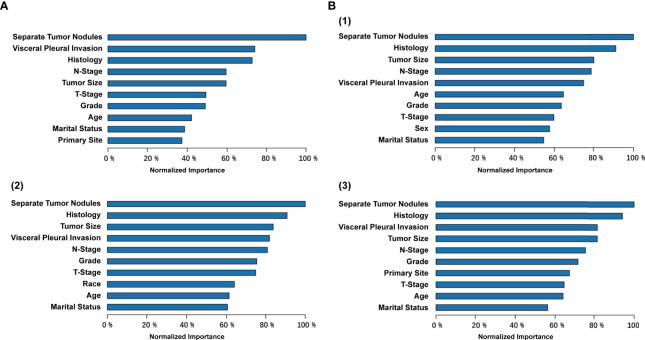
Variable importance from the artificial neural network (ANN) model for predicting **(A)** distant metastasis and **(B)** organ-specific metastasis [(1) bone, (2) brain, and (3) liver].

By applying ANN methods with variable importance measures, the importance of the 13 variables was standardized and the top 10 were showed in **Figure 5**. The most important variable was separate tumor nodules with 100% importance. The second important variable was visceral pleural invasion for distant metastasis, while histology for organ-specific metastasis. And the sex variable only appeared in bone metastases. Relatively, the race and insurance variable were less important in the whole cohort.

## Discussion

With the increasing incidence of distant metastasis of lung cancer, this field has gradually become one of the hot spots in clinical research (26–29). Our study suggested that distant metastasis was a risk factor for poor prognosis, and the median CSS for LC patients with bone metastasis, brain metastasis, liver metastasis and two or three metastases are 4 months, 4 months, 4 months and 3 months, respectively, which was similar to previous studies (28–32). Thus, early identification and diagnosis of distant metastasis is meaningful to improve prognosis and can assist clinicians in making therapeutic choices.

However, the cost of screening in an unselected population is considerable and the benefit is questionable, given the conflicting international screening guidelines and clinicians’ possible tendency to conduct investigations in excess of the recommended stage (14, 33–35). In this study, we developed a “two-step” ANN model for predicting synchronous organ-specific metastasis in LC patients. Our ANN model has high predictive power, with sensitivity of 0.906 for distant metastasis, 0.913 for bone metastasis, 0.925 for brain metastasis and 0.906 for liver metastasis. It can help predict the possibility of organ-specific metastasis in LC patients and alert high-risk patients for further investigation, which can provide clinicians with more accurate and personalized clinical decisions.

Previously, Zhou et al. used machine learning methods to analyze the distant metastasis possibility of lung cancer based on clinical and radiomic features (36). In this study, if only the features extracted from the CT image were used, the AUC was 72.84%. After combined with the patients’ clinical features, 89.09% could be achieved. The authors did not utilize ANN and included radiomic features, limiting direct comparison with our model. Recently, Liu et al. constructed a nomogram to predict bone metastasis of small cell lung cancer (SCL), which had a c-index of 0.745 in the internal validation set (30). Meanwhile, a multivariate model developed by Cacho-Díaz et al. was used to predict brain metastases of non-small cell lung cancer (NSCLC) and showed a predictive sensibility of 72% (27). Although the random forest classifier showed a good performance in predicting overall survival and the early response during radiotherapy in NSCLC, it performed unsatisfactorily in the predictions of our study (37, 38). Therefore, compared with traditional statistical models, our ANN model has superior performance.

In this study, we identified important features in the ANN model, with the top five including separate tumor nodules, visceral pleural invasion, histology, N-stage and tumor size, which were in line with the previous studies (27, 28, 30, 32, 36, 39, 40). Similar to our study, sex and N-stage were reported to be related to the occurrence of bone metastases (30, 32, 40). Interestingly, the correlation between larger tumor size and a higher risk of bone metastasis was uncertain (30, 39). And it was reported that age, sex, T-stage were independent predictors of brain metastasis (27, 28, 31, 41). Although the carcinoembryonic antigen (CEA) levels and epidermal growth factor receptor gene (EGFR) mutation status were associated with brain metastasis in patients with newly diagnosed NSCLC, we did not include these variables because they were not provided in the SEER database (27, 41).

This study should be considered in the context of several limitations. First, the study does not include an independent external cohort to validate the model, which is an important focus of future research. Nonetheless, we hope that the use of the SEER database, which accounts for about 28% of the United States population, will improve generalizability. Second, due to retrospective studies, the excluded missing data may lead to selection bias. Therefore, 25% of patients were randomly assigned to the testing group, which allowed for pseudo-prospective evaluation of our model and thus reduced bias.

In conclusion, despite the limitations, we developed and validated a novel ANN model for the prediction of synchronous organ-specific metastasis in patients with lung cancer. This ANN model may help clinicians to make individualized prediction and rational metastasis screening.

## Data Availability Statement

The original contributions presented in the study are included in the article/supplementary material. Further inquiries can be directed to the corresponding authors.

## Author Contributions

Conception and design: HG, Z-GW and LX. Administrative support: LX and XLD. Provision of study materials or patients: Z-YH and HG. Collection and assembly of data: Z-YH and HG. Data analysis and interpretation: Z-GW and HG. Manuscript writing: All authors. Final approval of manuscript: All authors.

## Funding

This study was supported by the National Natural Science Foundation of China (grant number 71874058).

## Conflict of Interest

The authors declare that the research was conducted in the absence of any commercial or financial relationships that could be construed as a potential conflict of interest.

## Publisher’s Note

All claims expressed in this article are solely those of the authors and do not necessarily represent those of their affiliated organizations, or those of the publisher, the editors and the reviewers. Any product that may be evaluated in this article, or claim that may be made by its manufacturer, is not guaranteed or endorsed by the publisher.
